# Linking Activity, Nutrition, and Child Health (LAUNCH): protocol for a longitudinal cohort study of children as they develop from infancy to preschool age

**DOI:** 10.1186/s12889-020-09023-7

**Published:** 2020-06-15

**Authors:** Russell R. Pate, Edward A. Frongillo, Kerry Cordan, Marsha Dowda, Alexander C. McLain, Myriam E. Torres, William H. Brown, Agnes Bucko, Emily R. Shull

**Affiliations:** 1 Department of Exercise Science, Arnold School of Public Health, University of South Carolina, 921 Assembly Street, Suite 212, Columbia, SC 29208 USA; 2 Department of Health Promotion, Education, and Behavior, Arnold School of Public Health, University of South Carolina, Discovery I, 915 Greene Street, 558, Columbia, SC 29208 USA; 3 Department of Epidemiology and Biostatistics, Arnold School of Public Health, University of South Carolina, Discovery I, 915 Green Street, Columbia, SC 29208 USA; 4 Department of Educational Studies, College of Education, University of South Carolina, 820 Main Street, Columbia, SC 29208 USA

**Keywords:** Physical activity, Sedentary behavior, Sleep, Diet, Weight status, Infancy, Early childhood

## Abstract

**Background:**

Physical activity is known to provide important health benefits in children ages 3 years and above, but little is known about the effects of physical activity on health in very young children under age 3. LAUNCH (Linking Activity, Nutrition, and Child Health) is a study designed to expand the body of knowledge on development of physical activity behavior and associations between physical activity and other health characteristics as children transition from infancy to preschool age.

**Methods:**

Physical activity and sedentary behavior will be measured objectively in young children over a period of 30 months. Each child will complete a measurement protocol at 6, 12, 18, 24, 30 and 36 months of age. The following factors will be measured at each time point: physical activity, sedentary behavior, anthropometric characteristics, and motor developmental status. Objectively-measured sleep behavior will be included as an optional component of the protocol. Parents will provide information on demographic factors, parenting behaviors, home and childcare characteristics, and the child’s dietary and sleep behaviors.

**Discussion:**

LAUNCH will employ a longitudinal study design and objective measures of physical activity, sedentary behavior and sleep in examining developmental trends for those characteristics in children between the ages of 6 and 36 months. Associations among physical activity, sedentary behavior, sleep, and weight status will be examined. Findings will inform public health guidance and intervention strategies for very young children.

## Background

Physical activity provides many important health benefits to children and adolescents. The U.S. Physical Activity Guidelines Advisory Committee concluded that higher levels of physical activity are associated with beneficial outcomes for bone health, weight status, cardiometabolic risk status, and brain health in school-age children and youth, ages 6 to 17 [1]. Further, the beneficial effects of physical activity on bone health and weight status extend to preschoolers in the 3- to 5-year-old age range [1]. Accordingly, the 2018 Physical Activity Guidelines for Americans include physical activity recommendations for both preschool and school-age children [2]. However, the Guidelines do not include physical activity recommendations for children under age 3, because the Guidelines Advisory Committee found that research on physical activity and health in infants and toddlers was too limited to support a conclusion [1]. The Committee’s report, which formed the scientific basis for the Guidelines, stated that future research should “develop valid instruments for measuring physical activity and examine the health effects of physical activity in very young children between birth and 2 years” [1].

Adiposity is a health outcome of great public health significance in children and adolescents, particularly in the economically developed nations of the world [3]. The prevalence of childhood obesity in the U.S. has increased dramatically since the 1980s [4], even in the youngest children [5]. In the most recent NHANES survey, 26% of 2- to 5-year-old children were overweight and 13% were obese [5]. This increased prevalence is likely the result of multiple changes in American society, including decreased physical activity and increased time spent in sedentary behavior [6]. The Committee concluded that strong evidence links lower levels of physical activity to increased likelihood of excessive gains in weight and adiposity in school-age children and preschool-age children [1]. Due to the limited number of studies available, however, the Committee was not able to study the relationship between physical activity and adiposity in infants and toddlers. Because a significant percentage of very young children demonstrate high weight to length ratios [7], better understanding of the factors that predispose young children to excessive weight gain is needed. Physical activity and sedentary behavior could be among these factors, and accordingly there is a need for research that examines the relationships between these behaviors and weight-related outcomes during early childhood [8].

The overarching goal of the LAUNCH study is to expand the body of knowledge regarding the relationship between physical activity and health in children as they develop from infancy to preschool age. A longitudinal study design and objective measurement of physical activity and sedentary behavior will be employed in addressing the following specific aims:

1. To describe physical activity and sedentary behavior in young children as they develop from infancy to preschool age.

- To describe the developmental trajectories for daily time spent in physical activity and sedentary behavior as children transition from infancy to preschool age.
- To determine the extent to which gender, race/ethnicity, and family socioeconomic status influence the developmental trajectories for physical activity and sedentary behavior.
- To determine the extent to which characteristics of children’s homes and childcare centers influence the developmental trajectories for physical activity and sedentary behavior.

2) To describe the longitudinal associations of weight status with physical activity and sedentary behavior as young children develop from infancy to preschool age.

- To determine the extent to which physical activity and sedentary behavior influence the developmental trajectories for weight status as children transition from infancy to preschool age.
- To determine the extent to which physical activity, sedentary behavior and dietary behavior exert independent influences on developmental trajectories for weight status.
- To determine the extent to which gender, race/ethnicity and family socioeconomic status moderate the influences of physical activity and sedentary behavior on developmental trajectories for weight status.

## Methods

### Study design

The LAUNCH study will use a longitudinal design to observe young children as they develop from infancy to preschool age. Each individual child will be measured at 6 time points. Measurements will begin at 6 months of age, and the protocol will be repeated at 6-month intervals across the observational period, ending at 36 months of age. That is, each participating child will complete a total of 6 measurement time points at 6, 12, 18, 24, 30, and 36 months of age. The funding period for the study is 12/05/2017–11/30/2022.

The following variables will be measured at each of the 6 time points: physical activity, sedentary behavior, weight, length, upper arm length and circumference, waist circumference, and motor developmental status. The child’s parent will also complete a questionnaire to assess demographics, social and physical environmental factors, gross motor milestones, and dietary behaviors at every time point. We anticipate that the responding parent will typically be the child’s biological mother, but in some cases it may be the father, grandparent or other adult caregiver. All questionnaires will be administered in English and Spanish, according to the parent’s preference. For children who attend a childcare center or preschool, the center director and a teacher will complete questionnaires, once a year, to assess center and classroom characteristics, and trained research staff will administer an Environment and Policy Assessment and Observation (EPAO) in each child’s classroom [9]. In addition, if the parent agrees to the optional sleep protocol, then objective sleep data will be collected at each of the 6 time points as well.

### Setting of the study

The LAUNCH study will be conducted in Richland and Lexington Counties, South Carolina. The population of Richland County is over 400,000, and in 2014 there were 4768 live births in the county [10]. The race/ethnicity distribution of residents in Richland County is 44% non-Hispanic white, 47% non-Hispanic black, 5% Hispanic, and 3% Asian. The population of Lexington County is over 260,000, and the race/ethnicity distribution of its residents is 75% non-Hispanic white, 16% non-Hispanic black, 6% Hispanic, and 2% Asian. Data collection will occur at an appropriate location of the parent’s choosing, including the child’s childcare center, the family’s home, the University of South Carolina, or a public library.

### Recruitment

Mother-child dyads will be recruited through childcare centers, pediatric offices, faith-based organizations, and at community events. This study aims to enroll 160 mother-child dyads, including both children who attend and do not attend formal childcare centers, with one-third of the sample being children who do not attend formal childcare centers at the time of their initial inclusion in the study. Richland County First Steps (RCFS), a non-profit that specializes in early childhood education and supports childcare centers and preschools, will assist with recruitment in its affiliated centers. In order to include children who do not attend a formal childcare center, project staff will also recruit parents and children at pediatric offices, faith-based organizations, and community events. Recruitment for the study will be completed by July 2020.

### Participant characteristics

Recruitment procedures for this study are designed to produce a sample that is approximately 38% non-Hispanic African American, 38% non-Hispanic White, 20% Hispanic, and 4% other races and ethnicities. To obtain a sample with the race/ethnicity distribution as described for Richland County above, Hispanic participants will be oversampled. The study aims to enroll an equal percentage of female and male children. Children will be excluded from the study if they were born before 37 weeks gestation or have a physical limitation that would invalidate accelerometry as a measure of physical activity.

## Measures

### Physical activity

Children’s physical activity will be assessed at each measurement time point using accelerometers (ActiGraph GT3X-BT model, Pensacola, FL). Accelerometers will be initialized prior to data collection and set to begin collecting data at midnight on the day of distribution. Children will be fitted with two accelerometers during each measurement session. Parents will be instructed to have their child wear the monitors on the right hip and the right ankle for a total of 7 days (except at night and during water-based activities). Monitors will be attached at the hip with an elastic belt and at the ankle with a sweatband. Both monitors will be worn at the 6-, 12-, and 18-month time points. Only the hip monitor will be worn at the 24-, 30- and 36-month time points, because at those ages the children are expected to be fully ambulatory. Trained and certified data collectors will provide parents and, as applicable, teachers and other caregivers, with detailed, verbal and written instructions to support compliance with the accelerometry procedures. Parents will be asked to place the monitor on their child shortly after waking up each morning and to remove the monitor each night before their child goes to bed.

Raw data will be downloaded from the devices and translated into 5- and 15-s epochs using Actilife software. Data will be stored by participant identification number in a private shared drive located on a secure, desktop computer. Any period of ≥60 min of consecutive zeros will be considered to be non-wear time and excluded from analyses. A valid 24-h day requires ≥16 h wear time. Observations with less than 3 days of valid data will be excluded from analyses. Since age-specific intensity cut-points have not yet been established for non-ambulatory children, no intensity cut-points will be utilized for the 6-month data. Rather, total daily physical activity will be defined as the counts per minute averaged over the 7 days of wear time. In ambulatory children, total physical activity minutes per hour and sedentary behavior minutes per hour will be determined through the use of age-specific cut-points [11].

Additional information will be obtained from the parent regarding the child’s physical activity behaviors. Parent surveys will ask about tummy time, time spent playing outdoors, participation in physical activity classes, time the child spent actively playing with the parent, and the time he or she spent actively playing with other children.

### Sleep

Information regarding children’s sleep behaviors will be assessed at each measurement time point. Sleep will be measured objectively using an actigraph (MicroMini-Motionlogger, Ambulatory Monitoring, Inc.). Participating in the sleep component of the protocol will be optional due to the substantial participant burden. The goal is to administer the sleep protocol at a minimum of three time points in at least 50% of the participating children. Actigraphs will be worn for 24 h per day for seven consecutive days and will only be removed for water-based activities. Children under the age of 2 years will wear the actigraph on the left ankle, while children 2 years old and older will wear the actigraph on the left wrist. Actigraphs will be placed in a small, soft, wrist band to reduce irritation, while still allowing the device to recognize wear-time.

Data will be collected in 60-s epochs downloaded from the monitors onto secure, desktop computers and analyzed using manufacturer-provided software (Action4, Ambulatory Monitoring, Inc.). The Sadeh et al. [12, 13] algorithm will be applied to the data to compute variables. The variables will include 24-h sleep duration, 24-h wake duration, nighttime sleep duration, nighttime wake duration, daytime sleep duration, daytime wake duration, and the number of nighttime awakenings. Daytime will be defined as 7:00 AM-7:00 PM, and nighttime will be defined as 7:00 PM-7:00 AM. Wear time will be established by using the LIFE channel provided by the software. A valid day will require either 20 h of wear time when measuring 24-h sleep, or 10 h of wear time when looking at daytime or nighttime sleep. Days that do not meet these decision rules will be marked as missing, and measurement time points with < 3 days of valid data will not be used in the analysis.

Additional information will be obtained from the parent regarding their child’s sleep habits. Parent surveys will ask about the child’s duration of sleep, sleeping arrangements, nighttime awakenings, and usual bedtime and naptime.

### Anthropometric measures

Children’s anthropometric measures will be assessed at each measurement time point. Using standard protocols, trained data collectors will measure the child’s weight, length, upper arm length and circumference, and waist circumference. For children unable to stand independently, length and weight will be measured using a Seca Digital Baby Scale and measuring rod (model 334; Chino, CA); for children able to stand independently, a Shorr board (Shorr/Weigh and Measure, LLC, Olney, MD) will be used to measure height, and a Seca scale (model 869; Chino, CA) will be used to measure weight. Age- and sex-specific weight-for-length percentiles and Z-scores will be calculated from weight and length according to the World Health Organization (WHO) growth charts [14]. For children 24 months of age and older, the age- and sex-specific body mass index (BMI) percentiles and Z-scores will be calculated according to the Centers for Disease Control and Prevention (CDC) growth charts [15].

### Motor development

Children’s motor development will be assessed at each measurement time point using the Peabody Developmental Motor Scales-2 (PDMS-2) [16]. Information will be gathered about each child’s motor milestones, reported by his or her parent. Two trained data collectors will administer and score the PDMS-2 at each measurement time point. Gross movement sub-tests will be administered according to standard age-specific protocols, including reflexes, stationary performances, locomotion, and object manipulation. A Gross Motor Quotient will then be calculated, indicating the sum of the child’s scores on 3 sub-tests. For children less than 1 year of age, the sub-tests will include reflexes, stationary, and locomotion; for children ages 1 to 3 years, the sub-tests will include stationary, locomotion, and object manipulation. Motor development performance will be reported as both a raw score and as an age-specific percentile.

Parents will report additional information regarding their child’s motor milestones through parent surveys. This will include the age in months when the child first rolled over, sat unassisted, began crawling, and began walking unassisted.

### Dietary behavior

Dietary behaviors will be assessed at each measurement time point. The parent will be asked to complete the Baby Eating Behavior Questionnaire [17] at the 6- month time point, which will provide information on the child’s appetite during the first 3 months of life, and the ECLS-B Parent Questionnaire (9-month version) [18], which will provide information on the child’s current and previous feeding behavior, including breast-feeding history. At the 12-, 18-, 24-, 30- and 36-month time points, the parent will complete the ECLS-B Parent Questionnaire (24-month version) [19], which will provide information on specific dietary behaviors, and the Multiple Indicator Cluster Survey (MICS) Dietary Intake survey [20, 21], which will provide information on foods consumed by the child on the previous day. At the 18-month and 36-month measurement time points, parent will also complete the Dietary Food Frequency Self-Administered Questionnaire, as developed for use in the National Children’s Study.

### Parent information

A parent survey will be administered at each measurement time point. Information regarding parent demographics, including age and race/ethnicity, will be collected at the first time point only. Information regarding marital status, parent education/employment (a proxy measure of socioeconomic status), pregnancy and delivery characteristics, and family makeup will be updated as necessary at each time point. Additional information will be collected at each time point, including mother’s parity, mother’s pre-pregnancy weight and gestational weight gain, whether the mother smoked cigarettes during pregnancy, and if the mother is pregnant with an additional child at the time of data collection. Mothers will also report their employment status (full-time, part-time, student, homemaker, disabled, retired, unemployed), the amount of time spent performing moderate and/or vigorous intensity physical activity over the past 7 days, and the average number of hours of daily screen media use.

### Home characteristics

Characteristics of the family home will be assessed at each measurement time point. Parents will provide information about the social and physical environment of their home via survey. This information will include the number of children living in the home, their ages and sex, and whether another adult lives in the home. Parents will also report the number of items of physical activity equipment located in the home, whether a television is located in the child’s bedroom, the total number of televisions found in the home, the average hours per day the child spends watching television and using other screen media devices, the average hours per day the child spends utilizing screen media that do not include a television, and the number of hours per day the child spends in confining equipment.

### Childcare center characteristics

Childcare center characteristics will be assessed yearly. Parents who report that their child attends childcare will also be asked to report the type of childcare utilized and the number of hours the child spends in childcare per day. Data collectors will perform a yearly, comprehensive evaluation of each center’s policies and practices related to nutrition and physical activity by administering director and teacher surveys, and by conducting direct observations.

#### Director survey

Center directors will be asked to provide information about their center’s characteristics and policies. The director survey will address global policies related to physical activity and nutrition as well as institutional characteristics. This includes information on the number of children enrolled in the center, the percent of children in each racial/ethnic group in the center, and information related to outdoor play and center nutrition policies.

#### Teacher survey

 Teachers will be asked to complete a survey about classroom practices related to physical activity and nutrition. The survey includes questions regarding classroom practices, including physical activity, sedentary behavior, meals and snacks, and teacher behavior and engagement, using the Go NAP SACC self-assessment instrument [22].

#### Environmental policy assessment and observations (EPAO)

Trained data collectors will conduct observations of the childcare environment using the EPAO [9]. The EPAO is a standardized instrument that assesses physical activity and nutrition in childcare environments. The EPAO will be completed by observing a single classroom during a full-day visit to a childcare center. Each center will receive scores for eight nutrition and eight physical activity subscales, as well as a total physical activity and total nutrition score.

## Statistical analysis

Initial analyses will include assessments of the frequency and distribution of all data elements and variables of interest. Demographic and population characteristics will be summarized by categories of the variables of interest to identify potential confounders using both descriptive statistics and formal comparisons, including t-tests for continuous variables or chi-squared tests for categorical variables. Final models will include all confounders found to change estimated regression coefficients for variables of interest by 10% or more [23]. Consistent with recent published guidelines on significance testing, the study will report 95% confidence intervals of estimated parameters [24].

The main analytic method for all aims will use a linear mixed-effects model. Each model will contain time, a random intercept, and possibly other random effects. We will consider using age in months as continuous (with possible transformations or spline smoothing) and categorical, with the best option being chosen by Akaike information criterion [25]. The analysis for Aim 1A will consist of characterizing the longitudinal trends in daily time spent in physical activity and sedentary behavior as children transition from infancy to preschool age, along with quantifying and determining the existence of between subject heterogeneity. The final model for Aim 1A will be used for the remaining aims including additional fixed effects based on the aim of the analysis. For Aims 1B and 1C, child-level and environmental-level characteristics will be added to the model to determine which characteristics are related to the overall status and trend of physical activity and sedentary behavior.

For Aim 2, we will implement a similar method as used for Aim 1 with weight status as the dependent variable. For Aim 2A, we will characterize the association of physical activity or sedentary behavior with weight status. The resulting model from Aim 2A will be used for Aim 2B with measures of dietary behavior added. This analysis will determine the association of dietary behavior with the status and trend of weight after adjusting for physical activity. For Objective 2C, a repeated-measures moderation analysis will be used to determine if gender, race/ethnicity, socioeconomic status, characteristics of home, or childcare center moderate the associations of the activity measures with weight status. To implement this analysis, models will include interaction terms, products of the potential moderator variables and the activity measures. A likelihood ratio test with a 0.05 significance level will be used to determine whether the interaction terms should be retained in the model. If this test is consistent with moderation, we will examine the individual tests to determine which moderator variable(s) drive the result.

Power calculations were run using R software and obtained via Monte Carlo simulation of the linear mixed model. The variances of the random effects were set to values that resulted in 0.2 correlation within subject’s outcome measures, and binary exposure variables were used throughout; continuous exposures result in higher power. The study will enroll 160 children; attrition was varied such that 60, 70, or 80% would complete the study. We used 5 and 6 longitudinal measurements, since the first measurement (i.e., at 6 months) may not be used for some analyses. All analyses used a significance level of 0.05. For Objective 1A the power to detect between-child variability was high (> 0.99) in all attrition and number of measurement settings. The results for Objectives 1B, 1C, 2A, and 2B (gender, race/ethnicity, socioeconomic status, characteristics of home or childcare center, weight status) were tested for small, moderate, and large effect sizes for analyses with 6 longitudinal measurements [26]. There is power > 0.99 for both medium and large effect sizes for all attrition and number of measurement settings; for small effect sizes power ranged 0.71–0.76 depending on the level of attrition. If 5 longitudinal measurements are used, the range of power for small effect sizes was 0.64–0.68 depending on the attrition. For Objective 2C, we ran similar power analyses that included an interaction term designating the moderation parameter. These simulation studies found that there was sufficient power (> 0.8) to detect medium and large effect sizes for the planned enrollment with 5 or 6 longitudinal measurements.

## Discussion

Additional efforts to meet participant recruitment goals and maintain participant retention will be implemented throughout the duration of the LAUNCH study. Specific recruitment procedures will be developed in order to yield a sample that reflects the race/ethnicity distribution of Richland County, including children from Latino families that may not be attending formal childcare centers. Procedures will be implemented by staff from South Carolina’s Consortium for Latino Immigration Studies and RCFS, which has considerable experience with the Latino community, to assist in the recruitment of Latino families. These efforts aim to recruit a population that is 20% Latino.

Additionally, specific procedures have been developed in order to maintain high participant retention for the duration of the study. Emails will be sent to participants by data collectors immediately after data collection as well as 6 weeks post-measurement at each time point, thanking them for their participation. Information regarding the next measurement time point will also included in this follow-up email. Emails will then be sent 1 month before the scheduled data collection time point in order to remind parents about the upcoming measurements. Parents will be asked to share any changes in contact information, addresses or childcare settings. If necessary, email communication will be done in Spanish by a Spanish-speaking staff member or data collector. Participants will receive a gift annually on the child’s birthday along with a LAUNCH birthday card. At the end of the study, children will receive a t-shirt along with a thank you card for participating. These will be in addition to a modest, financial incentive provided in the form of a gift card. Additional incentives will be provided for children who participated in the sleep component of the study. Teachers and childcare center directors will also receive gift cards for completing yearly surveys.

The LAUNCH study provides the opportunity to expand the body of knowledge regarding the relationship between physical activity and health during the first 3 years of life. In an effort to address limitations and current gaps in the existing literature, this study will use objective measurements of physical activity and a longitudinal study design to examine physical activity patterns and factors associated with those patterns from infancy to preschool age. Developing a full understanding of the effects of physical activity and sedentary behavior on changes in adiposity during early childhood is a critical research need, and findings from the LAUNCH study are likely to inform intervention efforts and recommendations for this age group.

## Data Availability

Not applicable.

## Abbreviations

LAUNCH: Linking Activity, Nutrition, and Child Health
NHANES: National Health and Nutrition Examination Survey
EPAO: Environment and Policy Assessment and Observation
RCFS: Richland County First Steps
WHO: World Health Organization
BMI: Body mass index
CDC: Centers for Disease Control and Prevention
PDMS-2: Peabody Developmental Motor Scales-2
ECLS-B: The Early Childhood Longitudinal Study, Birth Cohort
MICS: Multiple Indicator Cluster Survey
NAP SACC: Nutrition and Physical Activity Self-Assessment for Child Care
EPAO: Environmental Policy Assessment and Observations
AIC: Akaike information criterion
